# Translation elongation factor 1-alpha gene as a marker for diagnosing of candidal onychomycosis 

**DOI:** 10.18502/cmm.6.1.2503

**Published:** 2020

**Authors:** Keyvan Pakshir, Forough Farazmand, Farnoush Ghasemi, Hossein Mirhendi, Kamiar Zomorodian, Mahboobeh Kharazi, Ramtin Alborzi Pour, Hajar Golestani, Marjan Motamedi

**Affiliations:** 1 Department of Medical Mycology and Parasitology, School of Medicine, Shiraz University of Medical Sciences, Shiraz, Iran; 2 Basic Sciences in Infectious Diseases Research Center, Shiraz University of Medical Sciences, Shiraz, Iran; 3 Department of Medical Parasitology and Mycology, School of Medicine, Isfahan University of Medical Sciences, Isfahan, Iran; 4 Medical Mycology Laboratory, Razi Hospital, Tehran, Iran

**Keywords:** Candidal onychomycosis, Pan-Candida PCR assay, Translation elongation factor 1-alpha

## Abstract

**Background and Purpose:**

Culture-based identification methods have been the gold standard for the diagnosis of candidal onychomycosis. Molecular technologies, such as polymerase chain reaction (PCR) assays, can provide an alternative for the rapid detection of *Candida* species. The present study was conducted to investigate a pan-*Candida* PCR assay based on the translation elongation factor 1-alpha (*TEF-1α*) gene for the detection of the most prevalent pathogenic *Candida* species.

**Materials and Methods:**

For the purpose of the study, an optimized pan-*Candida* PCR primer pair was designed, and the target was amplified and sequenced. The analytical and clinical diagnostic performance of the designed primers was tested using 17 reference strains, 137 nail scrapings suspected of onychomycosis, and 10 healthy nail specimens.

**Results:**

The use of the universal *pan-Candida* primers designed on *TEF-1α* gene resulted in the successful amplification of a 270-base pair fragment in all *Candida* species tested, except for *C. glabrata,* and reacted neither with other fungi nor with *E. coli*. The sequence difference count matrix showed poor insertion/deletion differences (0-2 nt) among *Candida* species. Among 137 nail specimens, 35% (n=48), 30.7% (n=42), and 40.1% (n=55) of the samples were found to be positive by direct microscopy, culture, and pan-*Candida* PCR, respectively.

**Conclusion:**

Based on the findings, the PCR-based detection targeting the DNA *TEF-1α* gene is a rapid and simple procedure for the diagnosis of candidal onychomycosis directly from nail sample.

## Introduction

*Candida* species are common human commensal and important human yeast pathogens that cause a wide spectrum of diseases ranging from less severe superficial lesions to life-threatening systemic conditions in a vast spectrum of immunocompromised patients [1]. The incidence of infections due to *Candida* species has increased markedly as a result of a growing number of immunocompromised and critically ill patients [2]. *Candida* contains about 150 species. However, only a few of these species, including *C. albicans*, *C. tropicalis*, *C. glabrata*, *C. parapsilosis, C. dubliniensis, *and* C. krusei*, are implicated in human pathogenesis.

Superficial candidiasis may involve one or more mucocutaneous tissues, including the skin, mucous membrane, and nails [3]. Nail infections caused by *Candida* species known as candidal onychomycosis are normally associated with paronychia, onychia, or chronic mucocutaneous candidiasis. Further comprehensive studies have pointed out that *Candida* is the second most common etiology for onychomycosis after dermatophytes (tinea unguium) [4]. Conventional laboratory techniques for the diagnosis of candidal onychomycosis, such as direct microscopic investigation and mycological culture, are time consuming and laborious. Recent studies have shown that advances in molecular technologies are useful in detecting microbial infections without the need for microbial isolation [5, 6]. 

Nuclear ribosomal DNA (rDNA) regions, including ITS1 and ITS2, have been the most commonly used targets for the detection/identification of *Candida* species [7, 8]. Nevertheless, the description and characterization of new genetic markers for *Candida* species can clarify its taxonomy and might be helpful for detection/identification goals. The protein-coding genes (e.g., translation elongation factor 1-alpha [*TEF-1α*]) have been proven to be a powerful tool for the species delimitation of the taxonomically complex *Fusarium* species, considered an alternative to rDNA for species identification [9, 10]. Likewise, the gene is useful for developing robust phylogenetic inferences for other groups of pathogenic fungi, such as zygomycetes [11], dermatophytes [12], and *Aspergillus* species [13]. Since *TEF-1α* gene has not been used in *Candida* taxonomy and detection, our aim was to investigate *TEF-1α* as a new genetic marker to evaluate a pan-*Candida* PCR assay in the diagnosis of candidal onychomycosis. 

## Materials and Methods

***Standard strains and clinical specimens***

The specificity and sensitivity of the designed PCR system were determined against 17 standard strains of fungi. These strains included *C. albicans *(ATCC 5982, ATCC 2730, ATCC 562, ATCC 1912 and ATCC 64553), *C. tropicalis *(CBS 94 and ATCC 750), *C. krusei *(ATCC 6258), *C. glabrata *(ATCC 90030 and ATCC 863), *C. dubliniensis *(ATCC 8500 and ATCC 7987), *C. parapsilosis *(ATCC 4344 and ATCC 90018), *Cryptococcus neoformans* (ATCC 9011), *A. flavus *(ATCC 64025), *A. fumigatus *(ATCC 14110), and environmental isolates of *penicillium* *chrysogenum* and* Fusarium solan****i***. An standard isolate of *Escherichia coli* (ATCC 43894) was evaluated as a negative control. 

A total of 137 nail scrapings collected from patients who referred to the Medical Mycology Laboratory of Razi Hospital in Tehran [14] were used as clinical samples. Nail specimens from 10 healthy volunteers were used as negative clinical controls to verify the diagnostic specificity of the tests. All specimens were examined by both direct microscopy and culture, and a portion of each sample was stored at -20°C for use in the PCR assay. This research was approved by the Ethics Committee of the Shiraz University of Medical Science (IR.SUMS.REC.1398.020).

***Mycological examination***

A part of the samples was observed with 20% KOH, and the other part was inoculated onto three different points on Sabouraud dextrose agar (SDA) plate (Merck, Darmstadt, Germany) containing chloramphenicol (0.05 mg/l), with and without actidione (500 mg/l) (Merck, Darmstadt, Germany). The inoculated specimens were incubated at 28°C for 1-4 weeks. The culture plates were examined for fungal growth twice a week. 

***Primer design***

The sequences of ***TEF-1α*** in *Candida* species and other causative agents of onychomycosis, such as dermatophytes and non-dermatophytic molds, were downloaded from the National Center for Biotechnology Information (NCBI) (https://www.ncbi.nlm.nih. gov/pubmed/). The number of sequences for *Candida* species is limited in NCBI (a total of 13 *TEF-1α *sequences representing 5 species of *Candida*). Region of sequences that were conserved within *Candida* species but different from other fungi responsible for onychomycosis was chosen manually to design a pan-*Candida* primer pair (Table 1) considering the criteria specified by the Primer Biosoft [15] and optimized using the Multiple Primer Analyzer) https://www.thermofisher.com). The primers were also synthesized by the Bioneer Company (Bioneer, Korea).

***Preparation of genomic DNA***

DNA was extracted and purified from the fungal colonies using the boiling method [16]. Briefly, a small amount of fresh colony was suspended in 200 µL of distilled water, boiled at 95C for 15 min, and centrifuged for 4 min at 7,000 rpm; subsequently, the supernatant was used for PCR. For clinical specimens, DNA was extracted by means of a commercial kit (Yekta Tajhiz Azma, Iran) according to the manufacturer’s protocol after being treated by 500 μL of a digestion buffer (containing 400 mM Tris-HCL [pH=8], 60 mM EDTA [pH=8], 150 mM NaCl, and 1% sodium dodecyl sulfate) for 30 min and crushed for 3 min by a mechanical grinder.

***Polymerase chain reaction***

The PCR reactions were performed using 2X PCR Master Mix (Amplicon, Denmark), 0.5 μM of each primer, 4 μL of DNA template, and sufficient distilled water to reach a final volume of 25 μL. The amplification was accomplished using one cycle at 95°C for 3 min, as well as 35 cycles at 95°C for 30 sec, 55°C for 30 sec, and 72°C for 30 sec, followed by a final extension step at 72°C for 3 min. Negative (i.e., sterile distilled water) and positive (i.e., *C. parapsilosis* [ATCC 4344]) controls were included in each extraction run. Furthermore, 5 μL of the aliquots of the amplicons was electrophoresed using a 1.1% agarose gel in Tris-Borate-EDTA buffer (90 mM Tris, 90 mM boric acid, 2 mM EDTA, and pH of 8.3) and visualized under ultraviolet irradiation after ethidium bromide staining. A 100-bp DNA ladder was used as a molecular size marker.

***DNA Sequence Analysis***

The PCR product of standard *Candida* species examined in this study was purified and sequenced unilaterally with the forward primer on an automated DNA sequencer (ABI PrismTM 3500 Genetic Analyzer, Genetic Group) and edited with the Geneious (http://www.geneious.com) software. The sequences were compared with those in the Genebank database using the web-based software BLAST, (http://www.ncbi.nlm.nih.gov/BLAST). The sequence of each sample was subjected to ClustalW pairwise and multiple alignments using the MEGA6 software, and a phylogenetic tree was built using the maximum-likelihood algorithm with the Tamura-Nei parameter. 

**Table 1:** Standard and clinical strains of fungi sequences used in this survey for sequence analysis of translation elongation factor 1-alpha gene in silico studies

Species	Strain	*EF-1α* **accession no.**	**Size of ** *EF-1α* ** partial** sequence (bp)
*Candida albicans*	ATCC18804	DQ4472251	742
	ATCC 18804	KC5074361	929
	SC5314	XM7050562	1377
	SC5314	M299351	2369
	SC5314	M299341	2411
*Candida tropicalis*	ATCC 13803	DQ4472421	742
	MYA-3404	XM0025474801	1377
	MYA-3404	XM0025488371	1377
*Candida parapsilosis*	ATCC90018	DQ4472381	742
*Candida dubliniensis*	NCPF 3949	DQ4472291	742
	CD36-07890	XM002417390	1377
	CD36-22520	XM002418681	1377
*Candida glabrata*	ATCC 66032	DQ4472291	742
*Epidermophyton floccosum*	Clinical sample	KM6781911	732
	Clinical sample	KM6781881	732
	NBRC 9045	KM6780601	732
*Microsporum canis*	NRBC 9182	JN6629361	720
	CBS 130811	KM6780521	719
	mums138	MG3569341	648
*Microsporum gypseum*	CBS 174.64	KM6780691	733
	IFO 8228	KM6780571	748
	CBS 130820	KM6781611	748
*Trichophyton interdigital*	JCM 1891	KM6780581	766
	CBS 130788	KM6781421	768
	CBS 130940	KM6781731	767
*Trichophyton rubrum*	CBS 130927	KM6780551	737
	DSM 107606	MH8025051	644
	DSM 107668	MH8025061	624
*Trichophyton tonsurans*	CBS 130822	KM6780541	756
	mums73	MG3568841	667
	mums57	MG3568801	669
*Aspergillus fumigatus*	AX 2102 I	KJ4764101	873
	JCM 10253	KM9219611	674
	CBS 101640	KM9219681	612
*Aspergillus flavus*	CBS 573.65	KM9219761	663
	JCM 2061	KM9219751	684
	JCM2061	KM921975.1	684
*Aspergillus niger*	ITEM 7090	FN6654031	758
	ITEM 7496	FN6656571	758
	DUCC5023	KJ6382501	579
*Fusarium solani*	VI04903	FN6898261	691
	dq240	KR0255571	750
*Fusarium oxysporum*	NRRL 45915	FJ9854261	630
	NRRL 38544	FJ9854081	629
	NRRL 46589	FJ9854381	631
*Acremonium recifei*	CBS 188.82	KP0126471	763
	CBS 541.89	KP0126501	683
	CBS 220.84	KP0126481	554
*Scopulariopsis brevicaulis*	UTHSC 06-619	HG3803651	957
	UTHSC 06-1072	LM6525861	957
	UTHSC 09-1373	LM6525871	967
*Penicillium chrysogenum*	AC4503 II	KJ4764051	810
	AL0202	KJ4764061	921
	MOS731	KP0090001	990

***Analytical sensitivity of primers***

To determine the detection limit of this system, fungal inocula were prepared from culture for *C. albicans *(ATCC 5982). The inocula were adjusted to a turbidity of 0.5 on the McFarland scale, with approximately 1-5×10^6 ^CFU/ml, counted in a hemocytometer. The inoculum was diluted with sterile saline at a concentration of 10^1^-10^6^ CFU/ml. Subsequently, 200 μL of pool nail samples made from healthy nails added to each dilution. Commercial kit (Yekta Tajhiz Azma, Iran) was applied for DNA extraction from the nails treated with fungi. Eventually, PCR was performed on DNAs extracted from six serial dilutions and positive and negative controls.

***Statistical analysis***

The sensitivity, specificity, positive predictive value (PPV), and negative predictive value (NPV) were calculated for the pan-*Candida* PCR against the culture (as the reference method). The method of chi square test was used for categorical data. Statistically, the *P-value<.05* was considered as significant. The data were analyzed using SPSS software Version 22.0.

## Results

**Figure 1:**
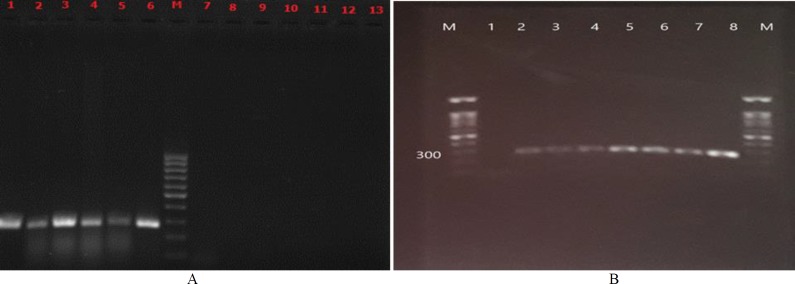
(A) Electrophoresis patterns of pan-*Candida* polymerase chain reaction using DNA template**; lane 1)*** Candida* ***albicans* (**ATCC 5982)**,** **lane 2) ***Candida tropicali***s (**ATCC 750)**, lane 3)** *Candida parapsilosi*s (ATCC 4344)**, lane 4)** *Candida albican****s*** (ATCC 64553)**, lane 5) ***Candida tropicalis* (CBS 94**), lane 6)** *Candida krusei* (ATCC 6258**), lane 7)** *Aspergillus* *fumigatus* **(**ATCC 14110)**, lane 8)** *Aspergillus flavus* (ATCC 64025**), lane 9)*** penicillium* *chrysogenum* (clinical isolate)**, lane 10)** *Fusarium solan****i*** (clinical isolate)**, lane 11)** *Cryptococcus neoformans* (ATCC 9011)*, *lane 12)* Escherichia coli* (ATCC 43894)*, *lane 13) **negative control (sterile distilled water),** and **lane M) a 100-bp molecular size marker;** (B) Determine the detection limit of this pan-*Candida* primers; lane 1) negative control (sterile distilled water), lane2) 2-7 nails treated with serially diluted standard strain (*C. albicans* (ATCC 5982)) at a concentration of 10^1^-10^6^ CFU/ml, lane 8 positive control (*C. parapsilosis* (ATCC 4344)) and lane M) a 100-bp molecular size marker

By aligning the ***TEF-1α*** gene sequences of most of the common *Candida* species, a highly conserved region was used for designing a new pan-*Candida* pair primer for PCR detection. The primers included CEFF: 5'-GGACAAAAACAGATTTGAA-3' as forward primer and CEFR: 5'-TTGCAATGGCAATCTCAAT-3' as reverse. The primers amplified an approximately 270-bp *Candida*-specific fragment in all tested *Candida* species, except for *C. glabrata* and reacted neither with other fungi nor with *E. coli* DNAs (Figure 1 left).

**Figure 2:**
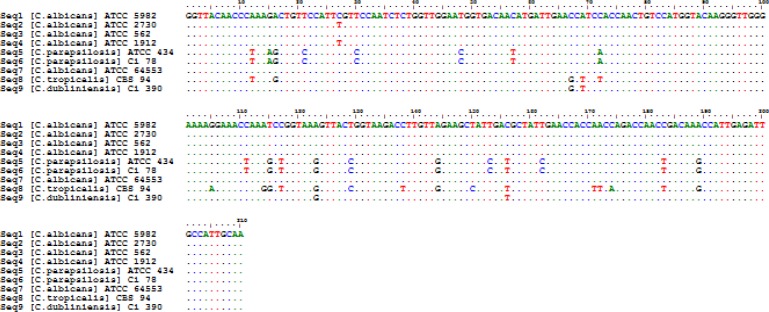
Multiple sequence alignments of translation elongation factor 1-alpha gene sequence from various *Candida* species sequenced in this study

The sequence difference count matrix showed poor intraspecies differences among the species belonging to *Candida* (0-2 nt insertions/deletions). Pairwise and multiple alignment rates were also evaluated among the sequences. The mean similarity of two sequences for 9 *Candida* isolates was obtained as 94.6%, and the overall similarity of the sequences was estimated at 86.6%. Figure 2 depicts the multiple DNA sequence alignment of *EF-1a* sequences in the most common pathogenic *Candida *tested in this study. The consensus nucleotide sequence data determined in this study were deposited in the GenBank, under the accession numbers of MK568517-MK568521 and MN231082-MN231085.

**Figure 3:**
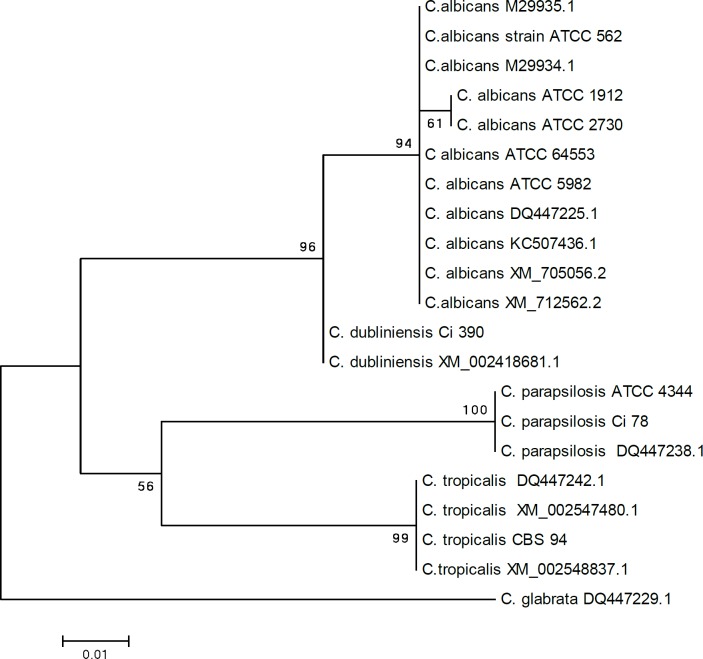
Phylogenetic analysis of elongation factor 1-alpha gene of 9 DNA isolates (standard and clinical isolates) of *Candida* species tested in this study and a number of sequences in this gene region related to *Candida* species in the gene bank (NCBI)

Phylogenetic tree of ***TEF-1α*** sequences in *Candida* species indicated that the nine strains tested in this study (i.e., *C. parapsilosis* clinical isolate Ci 78, *C. tropicalis* CBS 94, *C. dubliniensis* Ci 390, *C. albicans* ATCC 5982, *C. albicans* ATCC 2730, *C. albicans* ATCC 562, *C. albicans* 1912, *C. albicans* ATCC 4344, and* C. albicans* ATCC 64553) were most closely related with strains derived from the gene bank and shared a common branch. In the phylogenetic tree, the studied species were divided into two clades. Clad one consisted of two clusters of *C. albicans* and *C. dublinensis* and clad two contained three species of *C. parapsilosis*, *C. tropicalis*, and *C. glabrata* (Figure 3). 

For the determination of the analytical detection limit of the designed PCR reaction, serially diluted DNAs were assayed by pan-*Candida* PCR (Figure 1 right). According to the results, 10^1^ cells/mL was the minimum number for reliable detection. 

**Table 2:** Agreement of microscopy and Pan-*Candida* PCR methods with the gold standard method (culture)

		**Culture**		**Total**
		**True positive**	**True negative**	
**Microscopy**	Positive	n=42	n=6	n=48
	Negative	n=0	n=89	n=89
**Pan-** ***Candida *** **PCR**	Positive	n=35	n=20	n=55
	Negative	n=7	n=75	n=82
**Total**		n=42	n=95	n=137

PCR: polymerase chain reaction

Out of the 137 tested nail samples suspected of onychomycosis, 35% (n=48), 30.7% (n=42), and 40.1% (n=55) of the specimens were positive for yeast elements by direct microscopy examination, culture, and pan-*Candida* PCR, respectively (Table 2). 

The proportion of patients with positive pan-*Candida* PCR was higher than that of the patients with positive culture for *Candida* species (30.7% vs. 40.1%, respectively). This difference was statistically significant (*P<0.001*). Considering culture as the reference method to assess the performance of the molecular method used in this study, the PPV, NPV, specificity, and sensitivity were calculated as 63.3%, 91.4%, 78.9%, and 83.3% for pan-*Candida* PCR test, respectively. 

## Discussion

Rapid and accurate detection of *Candida* species in nail specimens is essential for the initiation of proper antifungal treatments. Among the routine methods, KOH preparation is easy to perform and economic; however, its sensitivity is inconsistent and influenced by such factors as inadequate sample volume, different sampling methods, and experience of the lab workers. Although fungal culture can identify specific pathogens, it takes a long incubation period and its sensitivity is much lower than that of KOH. In this regard, the false-negative rate of culture for the diagnosis of onychomycosis was approximately 30%, and the sensitivity was about 60% [17].

DNA-based diagnostic tests are increasingly being employed in the clinical microbiological laboratory for the detection of the etiological agent directly from the clinical specimens. The sensitivity of the detection molecular methods depends on various factors, one of the most important of which is the selection of the target DNA for amplification. Over the last two decades, the use of molecular diagnostic study for the detection of a specific sequence of *Candida* to investigate intra-species and inter-species differences has been reviewed [18, 19]. In such studies, when the main purpose was to design pan-*Candida* primers, the designed primer pair usually amplified common environmental contaminants, such as *Aspergillus*. The present study involved the design of a specific pan-*Candida* primer set in the ***TEF-1α*** gene where the primer sequences are conserved within *Candida* species but distinct from a variety of fungi other than *Candida* species and the bacterium* Escherichia coli*. The nucleotide sequence of ***TEF-1α*** gene encoding a part of the protein translation machinery appears to be consistently single copy. The only study investigating ***TEF-1α*** gene in *Candida* was conducted in 1990 by Sundstrom et al. to determine whether any of the characteristics of fungal ***TEF-1α*** proteins could be used to delineate the phylogenetic relationship of *C. albicans* with other fungi. Sequence analysis of the study showed the presence of two genes, called TEFI and TEF2, for ***TEF-1α*** in *C. albicans*. The coding regions of TEFI and TEF2 differed by only five nucleotides and encoded identical TEF-1α proteins of 458 amino acids [20].

This study represents the first application of a PCR-based diagnostic test for the most common *Candida* species using primers amplifying 270-bp region of the ***TEF-1α*** gene. Our results showed a great increase in the detection rate of *Candida* species using pan-*Candida* PCR (40.01%) over the direct microscopy (35%) and culture (30.7%). In the same vein, Khan et al. obtained more positive results by PCR than conventional methods (52.2% % vs. 21.2 %) [21].

In our study, 7 out of 42 samples which were positive in culture showed negative results in pan-*Candida* PCR. The DNA from these samples was diluted, and in another reaction, was exposed to pan-fungal primers (ITS1 and ITS4); however, the results stayed negative in the PCR. The positive and negative controls in all experiment runs ruled out the possibility of experimental error. The few negative results can be due to the fact that positive material was not included in the subsample set used for molecular testing. 

The pan-*Candida* PCR detected *Candida* in 20 samples identified negative by culture. This nonconformity that reduce the specificity (78.9%) and PPV (63.3%) of pan-*Candida* PCR has been linked rather to false-negative results of the culture method because PCR is more sensitive than culture methods and can detect all pathogenic fungi, both viable nonviable types.

Our pan-*Candida* primers facilitated the detection of DNA for all tested *Candida* species, except for *C. glabrata*. This finding is in the same line with the results obtained by Zhang et al. evaluating four pan-*Candida* primer sets by conventional PCR assays [22].

The lower limit of detection in this assay was 10^1 ^cells/mL which could be sufficient to find the low number of fungal elements causing candidal onychomycosis in a small volume of nail samples although this would require a highly efficient extracted template DNA. *Candida* DNA was detected in a few nail samples obtained from healthy volunteers, suggesting that this represented commensal fungi living on the nails. Accordingly, such methods as quantitative real-time PCR can be important for the appropriate evaluation of clinical samples containing both pathogenic and commensal fungi.

## Conclusion

As the results of the present study indicated, pan-*Candida* PCR assay showed good performance characteristics by increasing the detection rates of *Candida* species and drastically reducing the clinical turnaround time, compared to culture. However, it is required to perform further research using more types of standard isolates and larger clinical samples to define the practical value of such an approach in the mycology laboratory.
